# Tanshinol Alleviates Microcirculation Disturbance and Impaired Bone Formation by Attenuating TXNIP Signaling in GIO Rats

**DOI:** 10.3389/fphar.2021.722175

**Published:** 2021-07-14

**Authors:** Wenxiu Lai, Yulin Mo, Dongtao Wang, Ying Zhong, Lujiao Lu, Jiajia Wang, Liao Cui, Yanzhi Liu, Yajun Yang

**Affiliations:** ^1^ Department of Pharmacology, Guangdong Key Laboratory for Research and Development of Natural Drugs, Guangdong Medical University, Zhanjiang, China; ^2^ Department of Phamacy, Yuebei People’s Hospital, Shaoguan, China; ^3^ Department of Orthopedics and Traumatology, Nanning Hospital of Traditional Chinese Medicine, Guangxi University of Chinese Medicine, Nanning, China; ^4^ Department of Traditional Chinese Medicine, Shenzhen Hospital, Southern Medical University, Shenzhen, China; ^5^ Department of the Ministry of Science and Technology, Guangxi International Zhuang Medicine Hospital, Nanning, China; ^6^ Marine Medical Research Institute, Guangdong Medical University, Zhanjiang, China

**Keywords:** GIO, TXNIP (thioredoxin interacting protein), VEGF (vascular endothelial growth factor), β-catenin (CTNNB1), microcirculation dysfunction, bone metabolism, osteoporosis

## Abstract

Impaired bone formation is the main characteristics of glucocorticoid (GC)-induced osteoporosis (GIO), which can be ameliorated by tanshinol, an aqueous polyphenol isolated from *Salvia miltiorrhiza* Bunge. However, the underlying mechanism is still not entirely clear. In the present study, we determined the parameters related to microstructure and function of bone tissue, bone microcirculation, and TXNIP signaling to investigate the beneficial effects of tanshinol on skeleton and its molecular mechanism in GIO rats. Male Sprague-Dawley rats aged 4 months were administrated orally with distilled water (Con), tanshinol (Tan, 25 mg kg^−1^ d^−1^), prednisone (GC, 5 mg kg^−1^ d^−1^) and GC plus tanshinol (GC + Tan) for 14 weeks. The results demonstrated that tanshinol played a significant preventive role in bone loss, impaired microstructure, dysfunction of bone metabolism and poor bone quality, based on analysis of correlative parameters acquired from the measurement by using Micro-CT, histomorphometry, ELISA and biomechanical assay. Tanshinol also showed a significant protective effect in bone microcirculation according to the evidence of microvascular perfusion imaging of cancellous bone in GIO rats, as well as the migration ability of human endothelial cells (EA.hy926, EA cells). Moreover, tanshinol also attenuated GC-elicited the activation of TXNIP signaling pathway, and simultaneously reversed the down-regulation of Wnt and VEGF pathway as manifested by using Western-blot method in GIO rats, EA cells, and human osteoblast-like MG63 cells (MG cells). Collectively, our data highlighted that tanshinol ameliorated poor bone health mediated by activation of TXNIP signaling *via* inhibiting microcirculation disturbance and the following impaired bone formation in GIO rats.

## Introduction

Glucocorticoid (GC)-induced osteoporosis (GIO) is one of the most common secondary osteoporosis in clinical practice and may occur in people of all ages. In general, GIO is characterized by a marked impairment of bone formation, due to the decrease in osteoblast proliferation and activity (Taylor and Saag, 2019). To date, the therapeutic strategy for GIO depends mainly on antiresorptive drugs used for the treatment of postmenopausal osteoporosis, its characteristic feature distinguished from GIO. Teriparatide was an unique bone anabolic drugs approved by FDA owing to stimulating osteogenesis (Taylor and Saag, 2019). Notwithstanding, its application is limited by the vast expense and mode of injectable administration. Urgently, more focuses on the new findings involving in bone metabolism may be beneficial for the research and development of novel therapeutic approach for GIO treatment.

Bone vessels play multiple roles in the maintenance of bone homeostasis during physiological and pathological conditions besides participating in transport network. Bone microvascular niches supply oxygen, nutrients, and secrete cytokines required for bone tissue and the correlative cells (Sivaraj and Adams, 2016). Therefore, blood vessels can drive bone formation during development, repair, and regeneration (Xu et al., 2018; Chen et al., 2020). It is known that endothelial cells (ECs) releases crucial factors termed as “angiocrine signals” to regulate the behavior of neighboring cells in the varied bone microenvironment (Ramasamy et al., 2014; Sivan et al., 2019). Vascular endothelial growth factor (VEGF) is considered as an important modulating factor for bone remodeling in GC-induced osteoporosis (Pufe et al., 2003; Jiang et al., 2015). Since GC leads to the decrease of blood vessels and blood flow to the bone (Weinstein, 2010; Cui et al., 2012), microcirculation dysfunction has been named “blood stasis syndrome” in Traditional Chinese Medicine (TCM) (Liao, 2000), and might be considered as a new target for the treatment of GIO.

Thioredoxin-interacting protein (TXNIP), also known as vitamin D3-up-regulated protein (VDUP1), is an endogenous inhibitor of thioredoxin (Trx) to keep cellular redox-state homeostasis (Wu and Du, 2015). It was reported that the expression of TXNIP was abnormally increased in the bones of patients with Cushing syndrome (Lekva et al., 2012) and also high express in cells or in mice under the treatment of dexamethasone (Dex) (Wang et al., 2006; Reich et al., 2012). Further results indicated that TXNIP modulated osteoblast-mediated osteoclastogenesis by regulating the OPG/RANKL ratio to facilitate bone resorption (Lekva et al., 2012). Hence, limiting TXNIP directly or indirectly may be helpful to ameliorate osteoporosis (Mo et al., 2021; Yang et al., 2021). It was reported that high glucose-mediated overexpression of TXNIP induced a widespread impairment in endothelial cell (EC) function and survival by reducing VEGF production (Dunn et al., 2014). Moreover, hyperglycemia significantly up-regulated expression of TXNIP and down-regulated β-catenin pathway in endothelial cells (Yu et al., 2015). However, it’s still not clear to how TXNIP exerts the effect on bone metabolism, and how to regulate the canonical Wnt/β-catenin signaling pathway in the pathological process of GIO.

Tanshinol (D(+)β-3,4-dihydroxyphenyl lactic acid, also called danshensu), is the main bioactive component of *Salvia miltiorrhiza* Bunge, coinciding with “improve blood circulation and disperse stasis” based on the TCM theory. Our previous studies have highlighted the beneficial effect of tanshinol on improvement of bone formation *via* up-regulation of Wnt signaling pathway either in zebrafish, or in rats, and or in MSCs exposed to excessive GC (Cui et al., 2009; Luo et al., 2016; Yang et al., 2018). Our evidence also demonstrated that salvianolic acid B improves bone microcirculation and bone function in GIO rats (Cui et al., 2012). One other previous study indicated that salvianolate can inhibit TXNIP-mediated signaling pathway in a post-myocardial infarction model of rat (Qiu et al., 2018). As a consequence, the precise mechanism of tanshinol might involve in alleviating microcirculation disturbance, and then counteracting the damaged bone formation in GIO rats, in view of the fact that salvianolic acid B might be hydrolyzed into tanshinol (Guo et al., 2007). Particularly, whether tanshinol suppresses activation of the TXNIP signaling and rescues down-regulation of Wnt/VEGF cascade pathway in GIO rats remain need to be investigated. Based on the evidence mentioned above, the present study aims to comprehensively elucidate the anti-osteoporotic effect of tanshinol and its underlying mechanism in GIO rats and *in vitro*, and to provide a potential therapeutic insight for GIO.

## Materials and Methods

### Animal Experiments

4 month-old male Sprague–Dawley rats (396.4 ± 28.55 g, n = 48) were purchased from the Center of Experiment Animal of Guangxi Medical University (certificate of quality: SCXK (GUI) 2014-0002). The animals were housed in Guangdong Medical University animal facility according to the *Guide for the Care and Use of Laboratory Animals* of Guangdong Laboratory Animal Monitoring Institute. All experimental methods were approved by the Academic Committee on the Ethics of Animal Experiments of the Guangdong Medical University (Permit Number: SYXK (YUE) 2015–0147). All rats were fed with standard chow and free access to water with a 12 h light–dark cycle (25 ± 1°C). The rats were randomly assigned to the following four groups: Con, standard chow and CMC-Na (n = 12); Tan, Tanshinol 25 mg kg^−1^ d^−1^ (n = 12); GC, prednisone acetate 6 mg kg^−1^ d^−1^ (n = 12); Tan + GC (n = 12), GC plus tanshinol 25 mg kg^−1^ d^−1^ (n = 12). The drugs were administered for 16 weeks.

Thirty-six rats (9 rats per group) were injected subcutaneously with calcein (7 mg kg^−1^, Sigma, St. Louis, MO, United States) on day 13, 14, and day 3, 4 before sacrifice. All animals were sacrificed at the experimental end point. The left femurs were collected for evaluation of bone biomechanical properties and bone micro-architecture analysis. The proximal metaphysis of right tibias were explored to undecalcified section for bone histomorphometry analysis. The right femurs were prepared to detect expression of genes or proteins.

### Micro-CT, Bone Biomechanics and Morphometric Measurements

Bone trabecular microarchitecture of cancellous bone in the right proximal femur was determined by using Micro-computed tomography (Micro-CT, SCANCO vivaCT40, Basserdorf, Switzerland). In brief, the designated regions of femur to be scanned (18 μm/slice) were 1–4 mm distal to the growth plate-epiphyseal junctions. Trabecular 3D images were reconstructed by using micro-CT system. Then volume Bone Mineral Density (vBMD), Number of Trabeculae (Tb.N), Trabecular Thickness (Tb.Th), Trabecular Separation degree (Tb.Sp), Connectivity Density (Conn.D), and Bone volume fraction (BV/TV) were obtained from the system software.

For the histomorphometric detection, the tibia was fixed in 10% phosphate buffered formalin for 24 h, following by dehydrating in an ascending ethanol series, and then embedding undecalcified in methyl methacrylate. These tissues were cut into 5 mm sections to stain by toluidine blue for observing trabecular architectural property, including trabecular bone area (%Tb.Ar), Tb.Th, Tb. N, Tb.Sp, Number of Osteoclasts (Oc.N), active osteoclast surface (Oc.S/BS, %) and active osteoblast surface (Oc.S/BS, %), and 9 mm unstained sections for measuring the fluorescence labels in order to evaluate the indices of bone formation, such as Labeling Perimeter percentage (L.pm), Mineralization deposition rate (MAR), Bone Surface new Bone Formation Rate (BFR/BS) according to the two fluorescent labels. Histomorphometric assay was performed by using the Osteomeasure software (OsteoMetrics, Decatur, GA, United States).

Mechanical strength of long bone was measured by a three-point bending test by using the Testing machinery (MTS, Eden prairie, Minnesota, United States). The frozen left femurs were thawed at room temperature, and tested with a 1 mm indenter at a speed of 2 mm/min with a 15 mm span (L). Elastic load (N), fracture load (N), bending energy (N × mm) and stiffness (N × mm^2^) were obtained by calculation according to load-deformation curve.

### Serum Markers Assay

At the end of the experiment, rats that labeled by fluorescent were sacrificed by cardiac puncture under anesthesia with injection of 3% pentobarbital, serum was collected by centrifugation. The levels of serum of PINP (a marker of bone formation) and β-CTX (a marker of bone resorption) were determined by ELISA method.

### Bone Microvascular Perfusion Angiography

At the experimental end point, the remaining three rats in each group were subjected to MICROFIL® (Flow Tech Inc., CT, United States) perfusion. After anesthesia with chloral hydrate, the thoracic cavity was exposed, the left ventricle was cannulated to the aorta, and the right atrial appendage was cut. Sequentially they were perfused the mixture of heparin sodium saline, formalin and Microfil contrast medium. The cadaver specimens were kept overnight in a refrigerator at 4°C. After 24 h, sample of bilateral bone tissues of rats were collected, fixed in 10% formaldehyde for 24 h. After 1 month of decalcification, a Micro-CT (Viva CT 40, Scanco, Switzerland) was performed, a suitable threshold was set, and three-dimensional images of rat bone tissue were reconstructed and correlated. Quantitative analysis of the morphology of blood microvessels, such as the course of blood vessels, the way of interconnection, and the range of distribution, to obtain the ratio of microvessel volume (Mv.V/TV), microvessel connectivity density (Mv.conn.D), microvessel number (Mv.N), average microvessel thickness (Mv.th) and microvessel separation (Mv.Sp) related parameters, as described in detail (Cui et al., 2016).

### Cell Cultrue

Human osteosarcoma MG-63 cells (MG-63) and Human umbilical vein endothelial fusion cells (a permanent human cell line EA.hy926 cells, abbreviated to EA cells) were purchased from iCell (iCell Bioscience Inc., China). We chose the MG63 cell lines because it is no difference of bone metabolism between the MG-63 cell lines and the MC3T3-E1 or hFOB1.19 cell lines. The EA cells was derived by fusing human umbilical vein endothelial cells with the permanent human cell, line A549 (Edgell et al., 1983). MG cells and EA cells were cultured in MEM basic (Thermo Fisher, China) and DMEM High glucose (Thermo Fisher, China) in supplemented with10% fetal bovine serum (Thermo Fisher, China), at 37°C with an atmosphere of 5% CO_2_ and 95% humidity, respectively.

### Cell Scratch Test

EA cells were seeded in a 12-well plate at density of 5 × 10^4^ Cell/well. After the cells cultured for 24 h, the complete medium was added by containing different concentrations of Dex or Tan, each group has three multiple wells (1 ml/well). After culturing for 0, 6, 12, and 24 h, the cells were photographed for the analysis of the cell migration.

### Western Blotting Assay

For Western blotting, the right femur of rats in each group was ground into a fine powder while frozen with liquid nitrogen, and total protein was extracted from this powder using a total protein extraction kit. Cells were lysed by RIPA buffer containing complete protease inhibitor cocktail. Western blotting was performed as described in detail (Yang et al., 2016). There were biological replicates in the two lanes of every group both in GIO rats and in MG cells. The antibody recognizing TXNIP, VEGF, VEGFR2, β-catenin, GAPDH and α-Tubuling were purchased from Cell Signaling Technology (Beverly, MA, United States). The protein expression was monitored by the measurement of Chemiluminescence alterations using a FlourChem Q (Alpha Innotech., CA, United States) luminescent imaging system. The quantitative analysis of these images was performed using the Image-Pro Plus image software.

### Statistical Analysis

All experimental results were reported as mean ± SD, and *p* < 0.05, 0.01 and/or 0.001 were defined as the threshold of significance. Data was analyzed with the SPSS v20.0 statistical software package (IBM, Chicago, IL, United States). Samples were considered normally distributed if *p* > 0.05, 0.01 and/or 0.001 with Kolmogorov–Smirnov test. Heterogeneity of variance was accepted if *p* > 0.05, 0.01 and/or 0.001 and a Fisher least significant difference test was used for generating multiple comparisons between groups. Otherwise, the DunnettT3 test was used for such comparisons.

## Results

### Oral Administration of Tanshinol Attenuates Glucocorticoid-Induced Weight Loss

**FIGURE 1:**
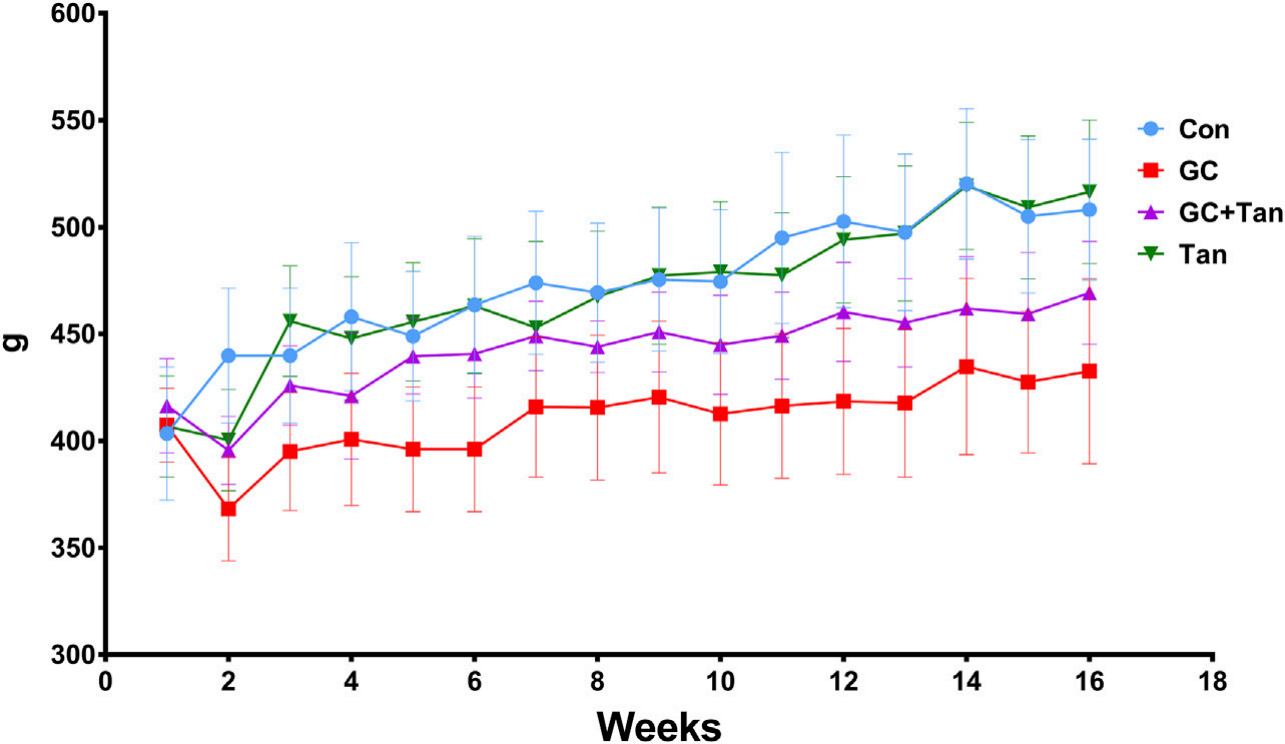
Changes of body weight during the experimental period. The body weight of all rats in each group was recorded each week for 16 weeks. The data are presented as the mean ± SD (n = 9). Note: Con, control group; GC, the group treated with prednisone acetate (6 mg kg^−1^ ⋅ d^−1^); GC + Tan, the group treated with GC plus tanshinol (25 mg kg^−1^ ⋅ d^−1^); Tan, tanshinol group.

All rats were flexible response and exhibited good appetite in each group. As shown in Figure 1, variation curve of body weight showed an ascending trend with age of rats during the experimental period, except for the second week. The body weight of rats in the GC group kept slowly increasing compared with the Con group, but body weight of rats both in the Con group and the Tan group maintained at the highest level. Interestingly, body weight of both the GC + Tan group was higher than those in the GC group.

### Tanshinol Rescues Glucocorticoid-Elicited Bone Loss and the Impaired Microstructure

**FIGURE 2:**
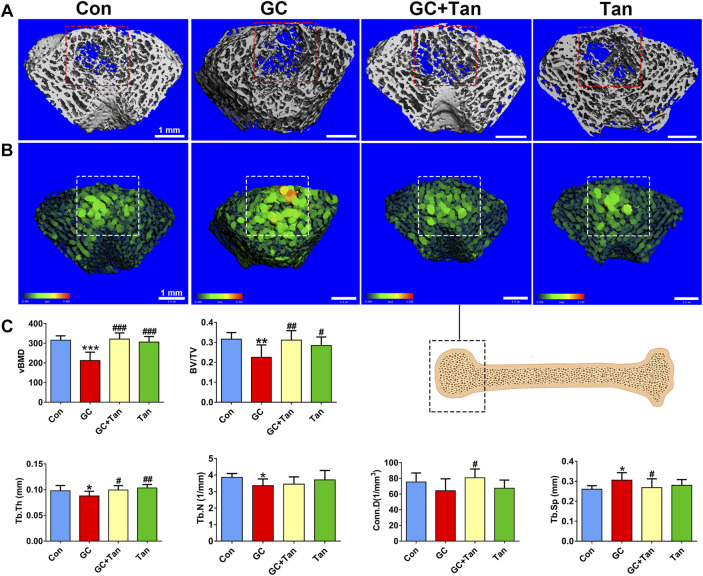
Effect of tanshinol on microarchitecture in GIO rats. At the experimental end point, the following measurements were carried out. **(A)** Micro-CT reconstruction of the trabecular part of distal femur. **(B)** Micro-CT reconstruction of the trabecular separation of distal femur. **(C)** Microarchitectural parameters of distal femoral spongiosa were measured by Micro-CT. Data are given as mean ± SD (n = 9). Note: ^∗^ *p* < 0.05, ^∗∗^ *p* < 0.01, ^∗∗∗^ *p* < 0.001 versus normal control (Con); ^#^ *p* < 0.05, ^##^ *p* < 0.01, ^###^ *p* < 0.001 versus GC treatment (GC).

To explore the protective effect of tanshinol on bone tissue of rats exposed to GC, we firstly scanned the cancellous bone by micro-CT and reconstructed the 3D-image of microstructure. The evidence demonstrated that the trabecular bone of rats in GC group showed impaired bone microarchitecture (Figures 2A,B), which were in accordance with the alterations of the main parameters, including volumetric bone mineral density (vBMD), bone volume to tissue volume (BV/TV) ratio, trabecular thickness (Tb.Th), trabecular number (Tb.N), connectivity density (Conn.D) and trabecular separation (Tb.Sp) (Figure 2C). Encouragingly, tanshinol exerted an apparent preventive effect on GC-triggered bone loss and impaired microarchitecture of rats (Figure 2C).

### Tanshinol Reverses Glucocorticoid-Initiated Dysfunction in Bone Metabolism and Poor Bone Quality

**FIGURE 3:**
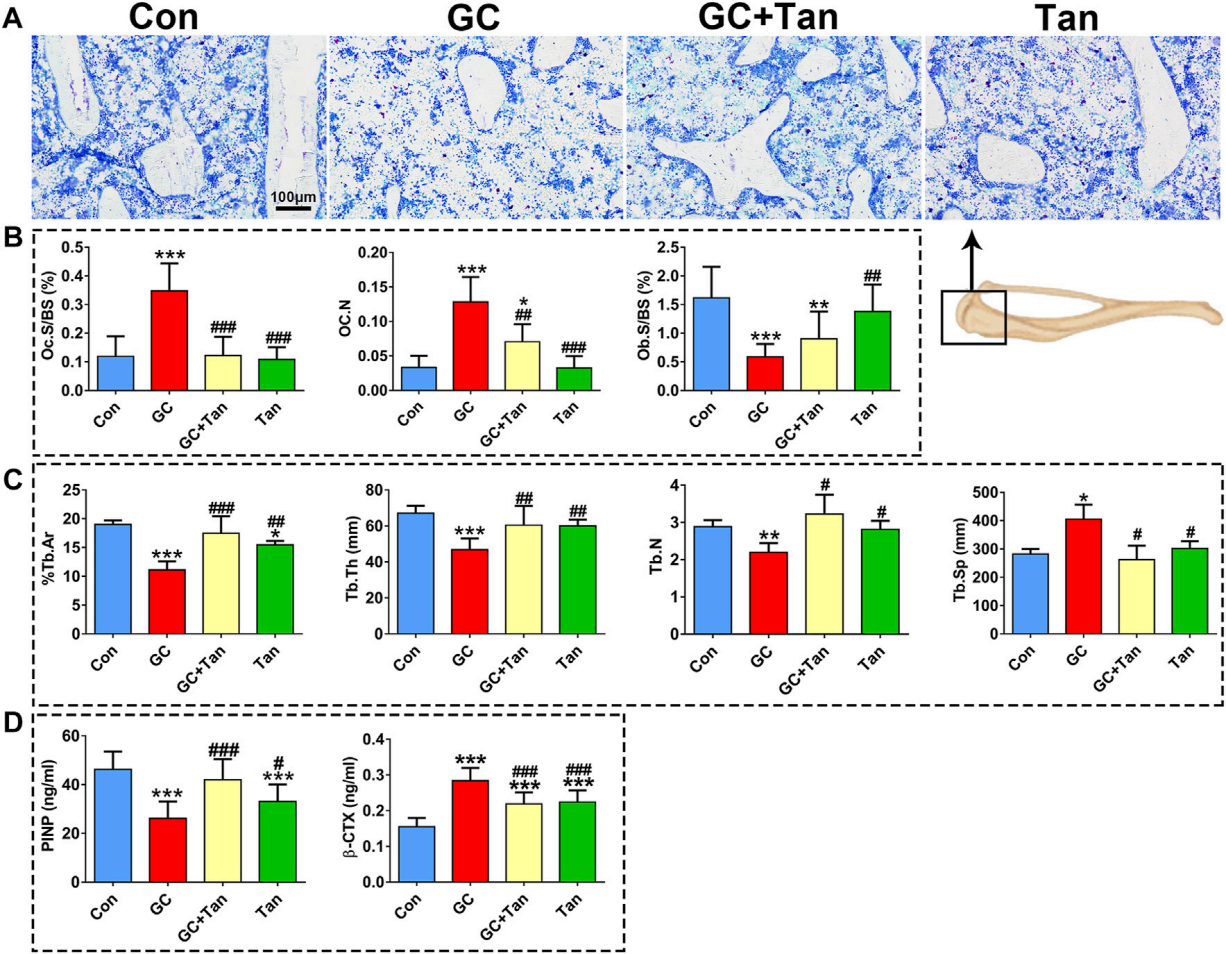
Effect of tanshinol on bone metabolism in GIO rats. **(A)** Toluidine blue staining of cancellous bone of the proximal tibia metaphysis. **(B)** Histomorphometric results of Oc.s/BS, OC.N, and Ob.s/BS of cancellous bone of proximal tibial metaphysis. **(C)** Static parameters of proximal tibial metaphysis in cancellous bone. **(D)** Serum markers of bone formation (PINP) and bone resorption (β-CTX) measured by using ELISA assay. The data are presented as the mean ± SD (n = 9). Note: ^∗^ *p* < 0.05, ^∗∗^ *p* < 0.01, ^∗∗∗^ *p* < 0.001versus Con; ^#^ *p* < 0.05, ^##^ *p* < 0.01, ^###^ *p* < 0.001 versus GC.

**FIGURE 4:**
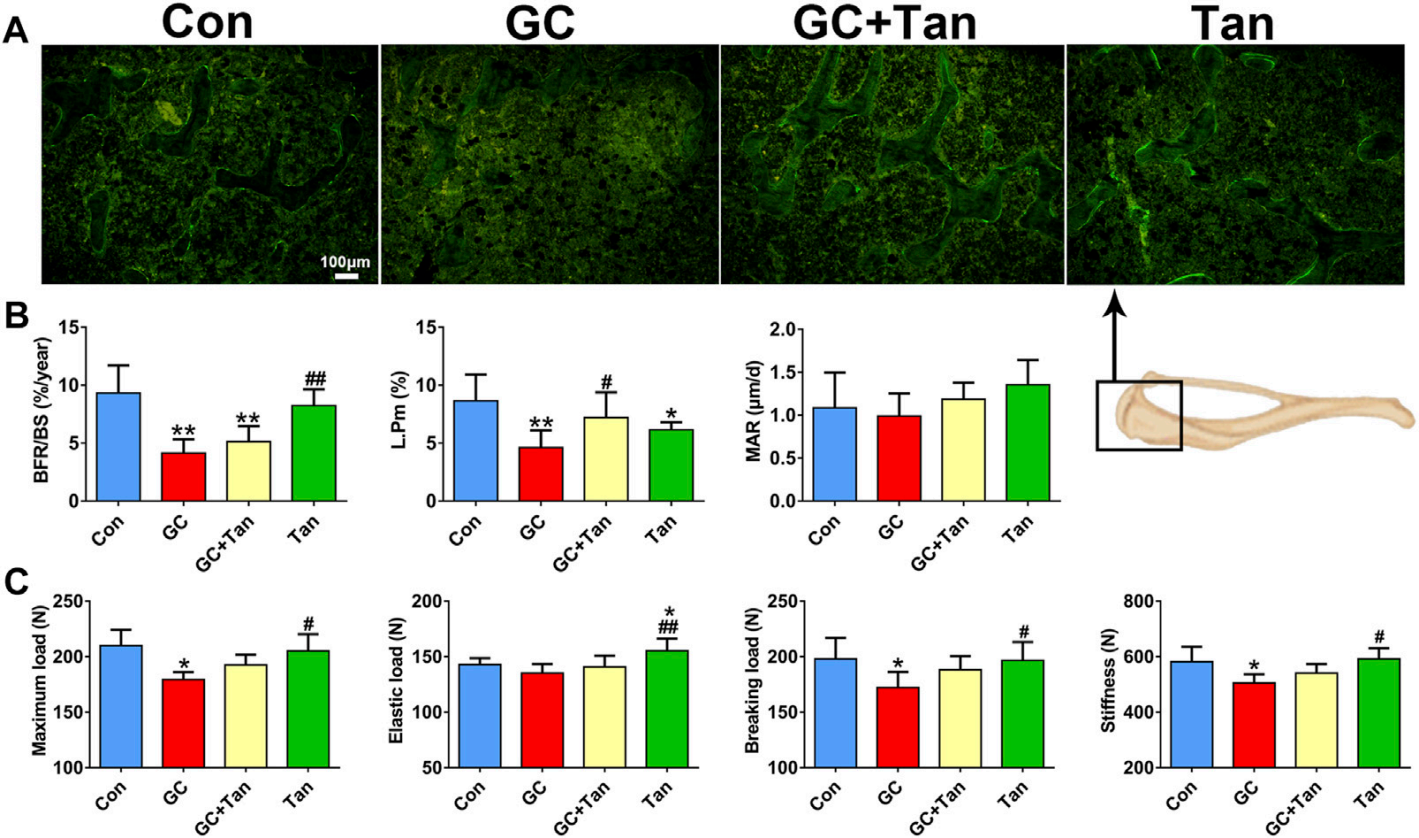
Effect of tanshinol on bone formation and biomechanical properties in GIO rats. **(A)** Representative fluorescent micrographs of dual calcein labeling in the tibia. **(B)** Histomorphometric quantitative analysis of dynamic parameters of %L.Pm, BFR/BS, and MAR used as key indicators of bone forming capacity in the tibia spongiosa. **(C)** Biomechanics characteristics of femur were determined by three-point bending assay. The data are presented as the mean ± SD (n = 9). Note: ^∗^ *p* < 0.05, ^∗∗^ *p* < 0.01, ^∗∗∗^ *p* < 0.001 versus Con; ^#^ *p* < 0.05, ^##^ *p* < 0.01, ^###^ *p* < 0.001 versus GC.

We next ask whether the preventive action of tanshinol on skeletal tissue associated with restoring the balance of bone metabolism in GIO rats. To begin with, the changes of %Tb.Ar, Tb.Th, Tb.N, and Tb.Sp in trabecular bone originated from the measurement of bone histomorphometry analysis were in line with those of the evidence measured by micro-CT analysis (Figure 2, Figures 3A,C). Furthermore, GC caused the increased number of osteoclasts and the following activity of mature osteoclasts, as well as the level of serum β-CTX (a bone resorption marker). Simultaneously, GC hampered the function of osteoblasts and bone formation indicated by the parameters including L.Pm (%), BFR/BS (%year) and MAR (μm/d), in accordance with the content of serum PINP (a bone formation marker). These deleterious alterations of bone remodeling can be rescued by tanshinol (Figures 3B,D, 4A,B). To testify whether tanshinol can exert beneficial influence on skeletal quality, femoral shaft samples were used for the three-point bending test. The evidence demonstrated that different kinds of parameters related to biomechanical properties were decreased in varying extent in GIO rats, and tanshinol exhibited a moderate trend towards ameliorating the deleterious effects of GC on biomechanical characteristics (Figure 4C). Collectively, tanshinol contributes to improve bone metabolism and bone strength in GIO rats.

### Tanshinol Abrogates Glucocorticoid-Elicited Disorder of Bone Microcirculation

**FIGURE 5:**
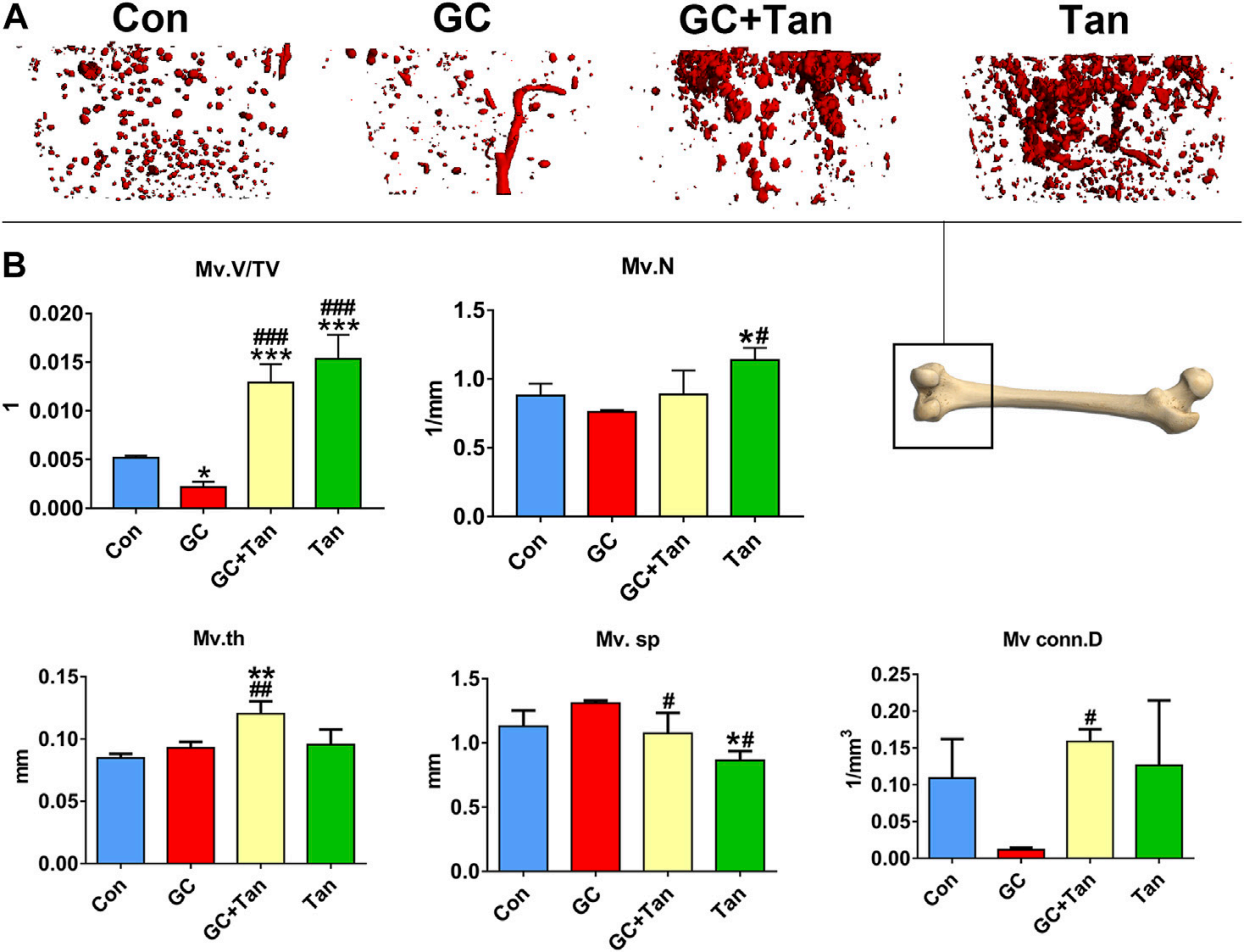
Protective effects of tanshinol on bone microcirculation in GIO rats. **(A)** Microfil microvascular perfusion imaging of microvascular structure of the distal femur. **(B)** Microvascular parameters of distal femoral were measured by Micro-CT. The data are presented as the mean ± SD (n = 3). Note: ^∗^ *p* < 0.05, ^∗∗^ *p* < 0.01, ^∗∗∗^ *p* < 0.001 versus Con; ^#^ *p* < 0.05, ^##^ *p* < 0.01, ^###^ *p* < 0.001 versus GC.

It is well-known that the vascular endothelium is considered as a mediator of bone health and disease (Prisby, 2017). We next examined the bone microcirculation by virtue of microfil microvascular perfusion imaging in rats treated with GC in the presence or absence of tanshinol. As shown in Figure 5, GC caused the decline of blood vessels in cancellous bone. In contrast, tanshinol could protect skeleton from GC-induced impairment and reduction of bone microvessels. Surprisingly, some parameters related bone microcirculation in GC + Tan group were higher than those in Con or GC group.

### Tanshinol Hampers Glucocorticoid-Provoked Up-Regulation of Thioredoxin Interacting Protein Signaling and Down-Regulation of Wnt/Vascular Endothelial Growth Factor Pathway

**FIGURE 6:**
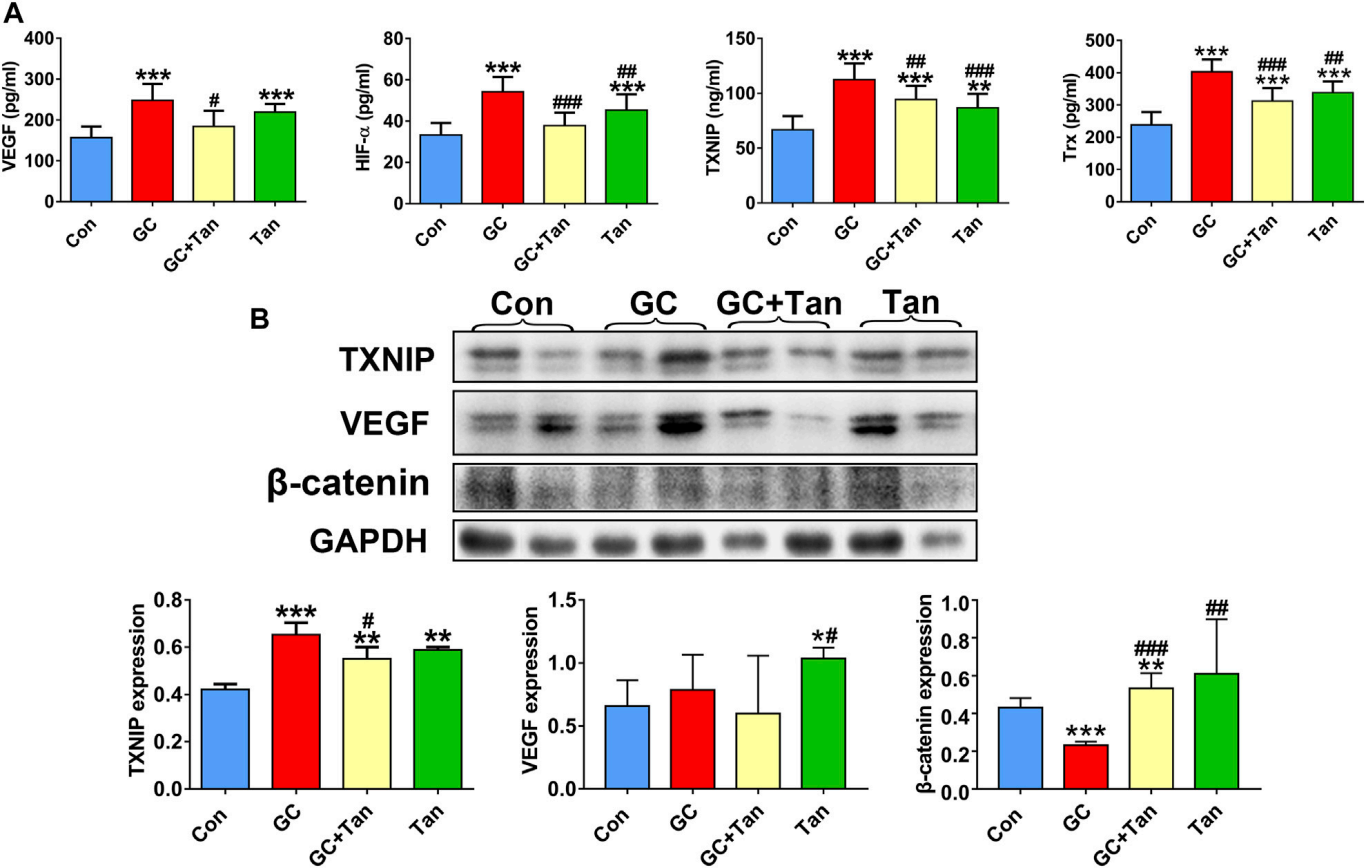
Tanshinol inhibits activation of TXNIP signaling and simultaneously rescues β-catenin/VEGF pathway in GIO rats. **(A)** Serum markers VEGF, HIF-α, TXNIP, Trx were measured using ELISA assay. **(B)** Expression of TXNIP, VEGF, β-Catenin (a key molecule of canonical Wnt signaling) in the bone tissue were measured by Western blot, and the two lanes of every group are biological replicates. Bars indicate mean ± SD of triplicate determinations. Note: ^∗^ *p* < 0.05, ^∗∗^ *p* < 0.01, ^∗∗∗^ *p* < 0.001versus Con; ^#^ *p* < 0.05, ^##^ *p* < 0.01, ^###^ *p* < 0.001 versus GC.

**FIGURE 7:**
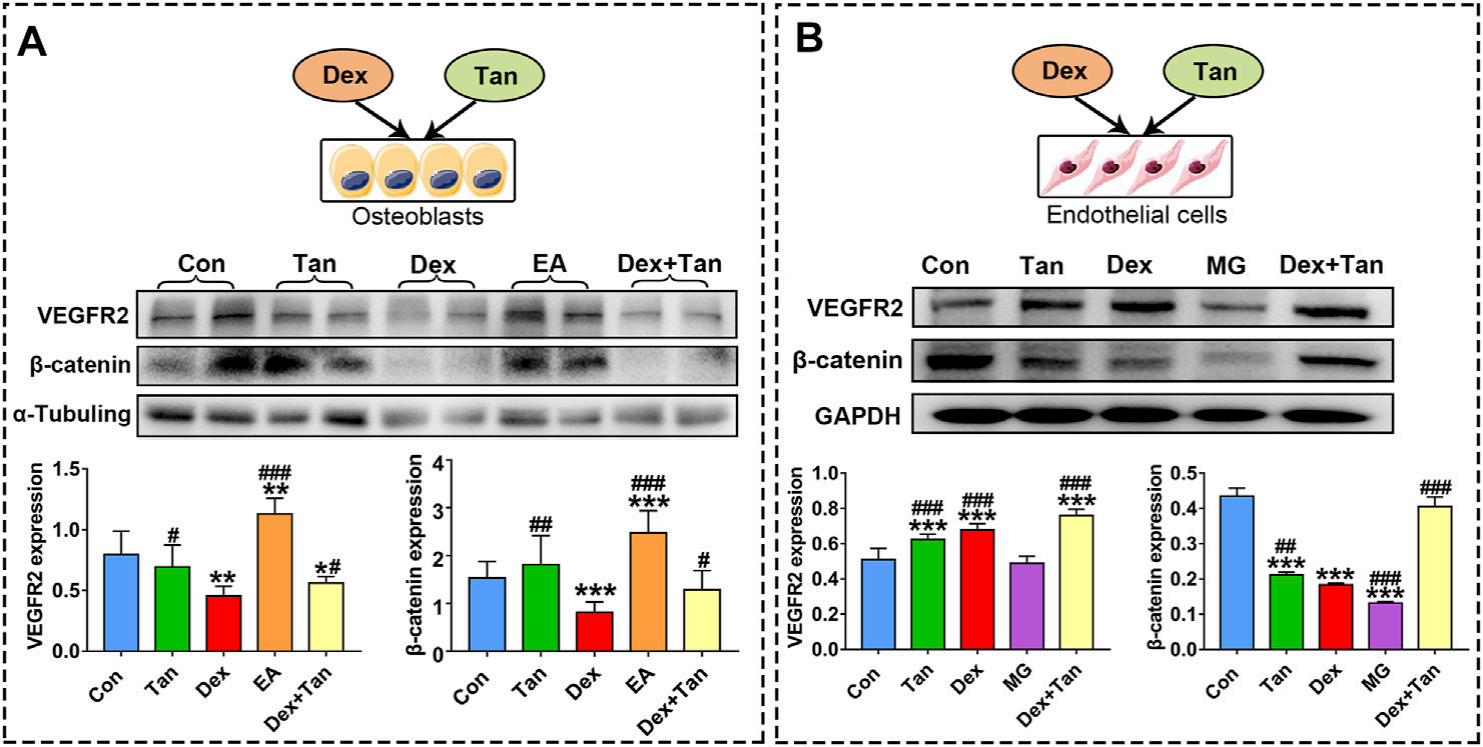
Effect of tanshinol on bone formation and blood vessel formation *in vitro*. Expressions of VEGFR2 (a key protein in angiogenesis) and β-catenin in MG cells and in EA cells, respectively. The two lanes of every group **(A)** are biological replicates. Bars indicate mean ± SD of triplicate determinations. Note: ^∗^ *p* < 0.05, ^∗∗^ *p* < 0.01, ^∗∗∗^ *p* < 0.001 versus Con; ^#^ *p* < 0.05, ^##^ *p* < 0.01, ^###^ *p* < 0.001 versus Dex.

**FIGURE 8:**
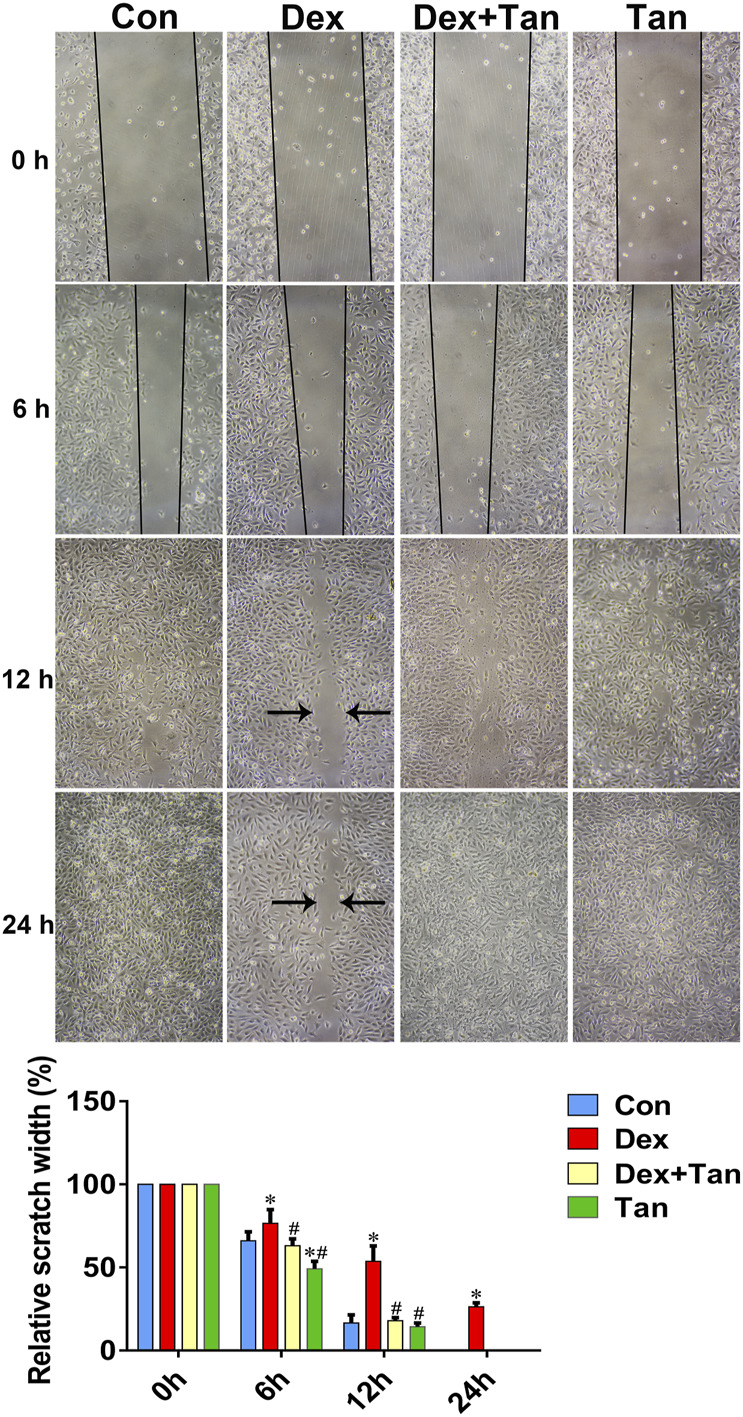
Tanshinol protects migration ability of EA cells against Dex. Effects of Dex or Tan on the migration of EA cells in 0, 6, 12, and 24 h was measured by scratch method respectively. Bars indicate mean ± SD of triplicate determinations. Note: ^∗^ *p* < 0.05 versus normal control (Con); ^#^ *p* < 0.05 versus dexamethasone treatment (Dex).

It has been previously demonstrated that GC can up-regulate TXNIP signaling in the bone tissue (Lekva et al., 2012), and TXNIP contributes to inhibition of canonical Wnt pathway (Hui et al., 2015) and VEGF pathway (Dunn et al., 2014). We further explored whether the osteoprotective action of tanshinol involved in counteraction of GC-induced activation of TXNIP signaling together with down-regulation of Wnt/VEGF pathway. The evidence of bone tissue acquired from the results of ELISA determination and Western blot assay proved that tanshinol reversed the increased expression of TXNIP, HIF-α, Txr and VEGF in GIO rats, and simultaneously restored the decreased expression of β-catenin (Figure 6), in line with the partial evidence collected from *in vitro* (Figure 7, Supplementary Figures 1, 2). As expected, GC leaded to inhibiting the ability of migration and tube formation of EA cells, and tanshinol could rescue the function within 24 h reaching to the extent of migration in normal control group (Figure 8 and Supplementary Figure 3).

Concerning the evidence that there was a cell-to-cell communication between osteoblast and endothelial cells (Veeriah et al., 2016), and a crosstalk between VEGF signaling and Wnt pathway (Wang et al., 2016), we next asked whether the supernatant fluid from MG cell culture can influence endothelial cells, and *vice versa*. The results demonstrated that only the supernatant fluid from endothelial cell culture can promote the expressions of VEGFR2 and β-catenin in MG cells (Figure 7). Moreover, tanshinol exerted a preventive effect on expressions of VEGFR2 and β-catenin in varying extent in the two cells (Figure 7). In brief, these evidences highlight the beneficial role of the tanshinol in angiogenesis and osteogenesis in GIO rats, in which may involve the down-regulation of TXNIP signaling and up-regulation of the Wnt/VEGF pathway.

## Discussion

**FIGURE 9:**
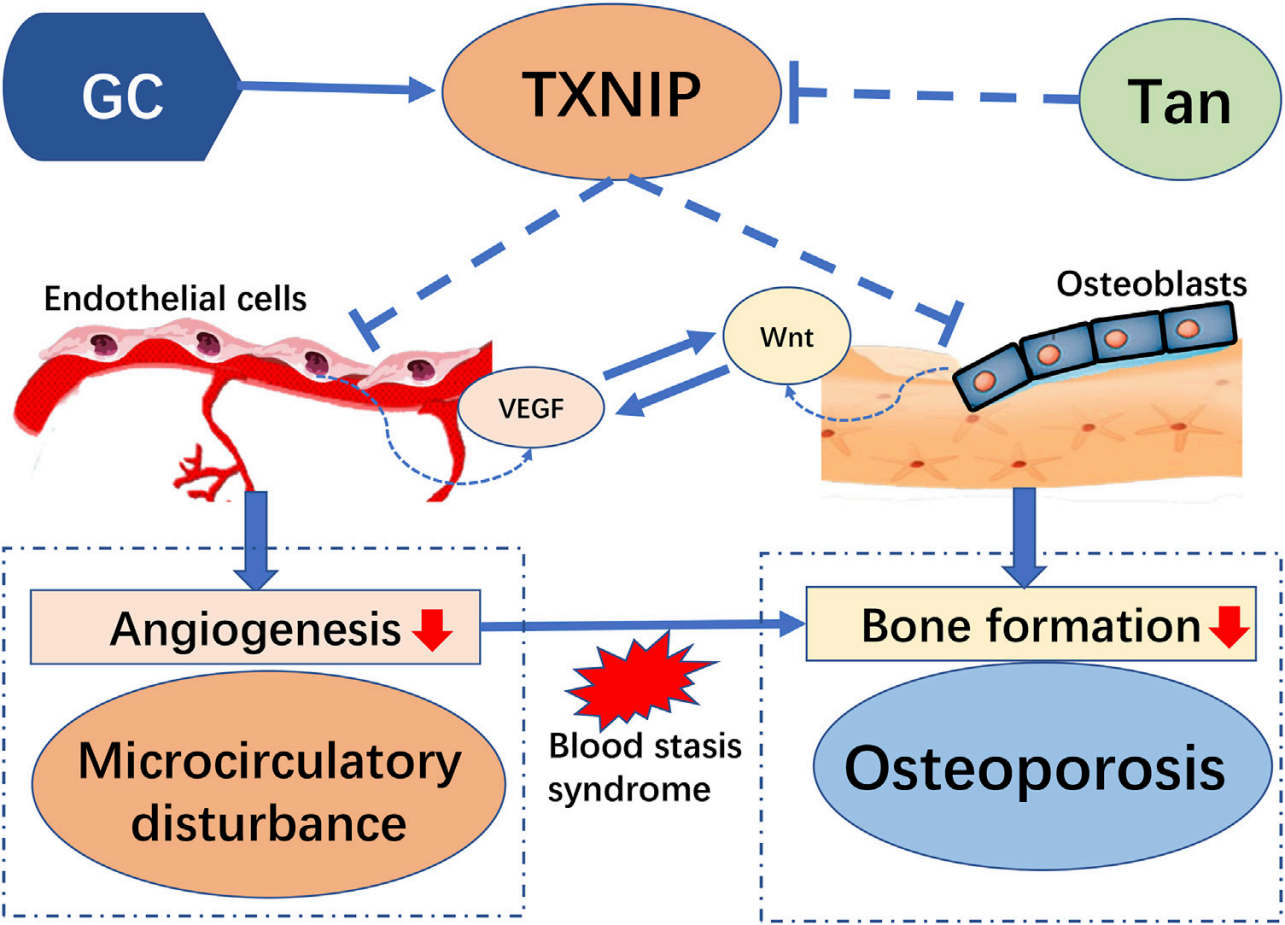
Model of the pathogenesis of GC-induced microcirculation dysfunction and impaired osteogenesis by TXNIP and its intervention of tanshinol: Long-term high-dose glucocorticoid can activate expression of TXNIP proteins. TXNIP down-regulates both VEGF and Wnt signaling pathways in vascular endothelial cells and osteoblasts respectively, contributing to inhibition of angiogenesis, followed by dysfunction of microcirculation in bone vessels, which causes blood stasis and decreased bone formation and eventually induced osteoporosis. However, tanshinol protects rats from GIO involving in the regulation of TXNIP/Wnt/VEGF cascade pathway.

Increasing documented evidences highlight the role of microcirculation dysfunction in the development and progression of GIO. Impaired bone microvasculature is a deleterious consequence owing to treatment with high doses and long-term administration of GC, and subsequently exert severe influence on function of bone formation (Peng et al., 2020). As illustrated in Figure 9, the present study provided a new finding to highlight the protective effect of tanshinol on bone tissue against the pathologic process of GIO, in accordance with the previous reports in our team (Cui et al., 2009; Luo et al., 2016; Yang et al., 2018). Here, we further confirmed that tanshinol exerts a protective effect on skeleton against the influence of GC relying on its inhibitory action on microcirculation disturbance and the following impairment of bone formation. Meanwhile, we demonstrated that the molecular mechanism of tanshinol underlying the anti-osteoporotic efficacy is related to regulation of the TXNIP/Wnt/VEGF cascade pathways. As tanshinol effectively relieved the impaired bone formation and improved bone quality, it seems to be a promising candidate reagent for the prevention and treatment of GIO.

It is well-known that bone metabolism is carried out from cradle to grave to maintain hardness and strength of skeleton. The pathophysiology of GIO is characterized by two distinct phases: an early rapid and transient phase followed by a slower, progressive phase (Compston, 2018). In the initial phase, GC triggers an imbalance between osteoclastogenesis and osteoblastogenesis during the remodeling, subsequently inducing an increase in the osteoclasts number and bone resorption and a decrease in the osteoblasts number and bone formation among patients on chronic steroid therapy (Taylor and Saag, 2019). Inevitably, the reduction in osteoblastic lineage cells leads to a corresponding reduction in both bone resorption and formation during long-term administration of GC, so-called low bone turnover (Chotiyarnwong and McCloskey, 2020). In this study, a high level of bone turnover was found in the GIO rats, indicating that bone resorption is higher than bone formation, and bone turnover in our study was still in the initial phase of GIO, according to the most commonly situation occurred in our previous studies and other reports (Cui et al., 2009; Cui et al., 2012; Yang et al., 2018; Zhang et al., 2018; Mo et al., 2021). Encouragingly, we found that tanshinol at least partially blocked the pathological progression of GIO, even some parameters related to bone metabolism in the Tan group restored to the similar extent like those in the Con group. Although it was no report that tanshinol exerted any effect on osteoclast differentiation and function, the evidences suggest that tanshinol exerts dual protective effects on bone tissue due to both blocking and reversing the pathological progression of GIO. In the light of the fact that tanshinol could meliorate GC-elicited osteoporotic bone loss, impaired bone microstructure, and poor bone strength by restoring the balance between bone formation and bone resorption, we assumed that tanshinol has a good application prospect for prevention and treatment of GIO in the future.

TXNIP has attracted considerable attention regarding drug development owing to its multiple functions and involvement in metabolic disorders, inflammation, neurodegenerative disorders as well as cancer (Qayyum et al., 2021). Generally, TXNIP is considered as a vital regulator in response to oxidative stress relying on interacting with reduced thioredoxin (Trx),and further blocking its potential for scavenging reactive oxygen species (Alhawiti et al., 2017). Recently, it was found that *TXNIP* gene highly expressed in the patients with endogenous Cushing’s Syndrome (Lekva et al., 2012). Our previous evidence further revealed that TXNIP elicited bone loss by promoting the excessive mitochondrial oxidative phosphorylation under conditions of oxidative stress induced by GC, which can be blocked in the TXNIP knockout mice (Mo et al., 2021). Several preclinical and clinical evidence related to diabetes mellitus supported the TXNIP-specific inhibitors for the development of new promising agents (Qayyum et al., 2021), such as Verapamil (Khodneva et al., 2016), Taurine (Gondo et al., 2012), and SRI-37330 (Thielen et al., 2020). It was reported that TXNIP pathway could be inhibited by a salvianolate injection (Qiu et al., 2018), the main water-soluble bioactive compounds of *Salvia miltiorrhiza* bunge. As one of the bioactive water-soluble components, tanshinol exerts exactly aforementioned protective effect on osteogenesis, in line with our previous studies (Cui et al., 2009; Yang et al., 2013; Luo et al., 2016; Yang et al., 2018). Therefore, there is a need to elucidate whether tanshinol protects bone function from injury evoked by long-term excessive use of GC *via* regulating the TXNIP pathway in GIO rat. Our present data showed that tanshinol contributed to suppressing the up-regulation of TXNIP signaling in GIO rats. That might be the underlying mechanism of tanshinol for treating GIO.

It is believed that VEGF secreted from osteoblasts can trigger signaling responses in different cell populations expressing VEGF receptors (VEGFR), including the endothelial cells and varied bone cells, such as osteoblasts and chondrocytes (Wang et al., 2007). In a variety of pathophysiological conditions, reduced osteoblasts may reduce the expression of hypoxia inducible factor 1a (HIF-1α) and the production of VEGF, thereby adversely affecting the vasculature (Sivaraj and Adams, 2016). As a target gene for HIFα, TXNIP is required for VEGF-mediated VEGFR2 activation and angiogenic response in hypoxia-induced abnormal angiogenesis (Abdelsaid et al., 2013). Similarly, we found here that the express of VEGF was high in the bone tissue of GIO rats and the express of VEGFR2 in EA cells were also at a high level exposed to Dex. In contrast, Dex could lead to a significant decrease in express of VEGFR2 in MG cells. These results suggest that GC-induced angiogenic response mainly occurs within endothelial cell. Additionally, a distinct finding was showed that the supernatant fluid from EA cell culture stimulated an obvious elevation of VEGFR2 express in MG cells, and the supernatant fluid from MG cell culture had little effect on endothelial cells. The discrepancy of this opposite evidence between the previous results and partial ours may be due to the different mechanisms in different experimental design. However, it is worthy of further study on the precise relationship between osteoblasts and endothelial cell in the bone tissue. Based on biomarker expression and functional characteristics, an endosteal type H capillaries who couples angiogenesis to osteogenesis was found to reduce strongly in bone of aged animals, contributing to bone loss (Kusumbe et al., 2014). As a matter of fact, GC can cause bone senescence (Pignolo et al., 2019). These evidence from literatures published may help explain the present result partially. In the present study, tanshinol could down-regulate the excessive expression of VEGF in the GIO rats, while almost no significant effect against GC was observed in the both cells. Further studies need to prove the findings that the preventive effect of tanshinol on bone microcirculation disturbance links to the type H capillaries.

Generally, osteoblastogenesis is mediated by the Wnt/β-catenin signaling pathway. This study validated that GC inhibit Wnt pathway both *in vitro* and *in vivo*, which can be reversed by tanshinol, consistent with our previous studies (Yang et al., 2013; Yang et al., 2018). In our previous study, KLF15, a novel identified glucocorticoid receptor (GR) target gene, exerted an inhibitory effect on Wnt pathway in osteoblast (Yang et al., 2018). However, whether a connection between TXNIP and KLF15 lied in osteoblasts and their interaction contributed to regulation of Wnt pathway remains to be elucidated. It was reported that endothelial cells supported osteogenesis of osteoprogenitor cells via up-regulation of Wnt signaling by activating β-catenin expression (Grellier et al., 2009; Maes et al., 2010), and that both β-catenin dependent and independent Wnt signaling pathways can control angiogenesis in EC cells (Phng et al., 2009; Korn et al., 2014). In the present study, the supernatant fluid from EA cell culture promoted the express of β-catenin in MG cells, but the supernatant fluid from MG cell culture exerted inhibitory effect on β-catenin in endothelial cells. Although an enormous amount of research efforts involved in the effect of endothelial cells on osteoblasts, the precise mechanism of osteoblasts on endothelial cells is still unclear. We have planned to confirm the findings mentioned above with the help of gene-conditioned knockout mice, rat’s primary osteoblast and endothelium cell in the next study. Collectively, the evidence highlights the complexity of molecular mechanism underlying tanshinol on osteoprotective effect in GIO rat.

## Conclusion

Our study demonstrated that tanshinol ameliorated microcirculation dysfunction and impairment of bone formation, revealing a significant osteoprotective effect as evidenced by the down-regulation of TXNIP signaling. However, it was not adequate signaling molecule to be detected in our study for explaining the relationship between tanshinol and the changes of signal transduction in the GIO model. Further experiments are needed, such as adding TXNIP inhibitors or the *TXNIP* knockout model of mice, to go deeper into the role of the TXNIP signaling pathway in the protection of tanshinol on GIO mice.

## Data Availability

The original contributions presented in the study are included in the article/Supplementary Material, further inquiries can be directed to the corresponding authors.

## References

[B1] Abdelsaid M. A. Matragoon S. El-Remessy A. B. (2013). Thioredoxin-interacting Protein Expression Is Required for VEGF-Mediated Angiogenic Signal in Endothelial Cells. Antioxid. Redox Signaling 19 (18), 2199–2212. 10.1089/ars.2012.4761 PMC3869450 23718729

[B2] Alhawiti N. M. Al Mahri S. Aziz M. A. Malik S. S. Mohammad S. (2017). TXNIP in Metabolic Regulation: Physiological Role and Therapeutic Outlook. Cdt 18 (9), 1095–1103. 10.2174/1389450118666170130145514 PMC5543564 28137209

[B3] Chen J. Hendriks M. Chatzis A. Ramasamy S. K. Kusumbe A. P. (2020). Bone Vasculature and Bone Marrow Vascular Niches in Health and Disease. J. Bone Miner Res. 35 (11), 2103–2120. 10.1002/jbmr.4171 32845550

[B4] Chotiyarnwong P. McCloskey E. V. (2020). Pathogenesis of Glucocorticoid-Induced Osteoporosis and Options for Treatment. Nat. Rev. Endocrinol. 16 (8), 437–447. 10.1038/s41574-020-0341-0 32286516

[B5] Compston J. (2018). Glucocorticoid-induced Osteoporosis: an Update. Endocrine 61 (1), 7–16. 10.1007/s12020-018-1588-2 29691807 PMC5997116

[B6] Cui L. Li T. Liu Y. Zhou L. Li P. Xu B. (2012). Salvianolic Acid B Prevents Bone Loss in Prednisone-Treated Rats through Stimulation of Osteogenesis and Bone Marrow Angiogenesis. PloS one 7 (4), e34647. 10.1371/journal.pone.0034647 22493705 PMC3321026

[B7] Cui L. Liu Y.-y. Wu T. Ai C.-m. Chen H.-q. (2009). Osteogenic Effects of D(+)β-3,4-dihydroxyphenyl Lactic Acid (Salvianic Acid A, SAA) on Osteoblasts and Bone Marrow Stromal Cells of Intact and Prednisone-Treated Rats. Acta Pharmacol. Sin 30 (3), 321–332. 10.1038/aps.2009.9 19262556 PMC4002398

[B8] Cui Z. Crane J. Xie H. Jin X. Zhen G. Li C. (2016). Halofuginone Attenuates Osteoarthritis by Inhibition of TGF-β Activity and H-type Vessel Formation in Subchondral Bone. Ann. Rheum. Dis. 75 (9), 1714–1721. 10.1136/annrheumdis-2015-207923 26470720 PMC5013081

[B9] Dunn L. L. Simpson P. J. L. Prosser H. C. Lecce L. Yuen G. S. C. Buckle A. (2014). A Critical Role for Thioredoxin-Interacting Protein in Diabetes-Related Impairment of Angiogenesis. Diabetes 63 (2), 675–687. 10.2337/db13-0417 24198286 PMC3900553

[B10] Edgell C. J. McDonald C. C. Graham J. B. (1983). Permanent Cell Line Expressing Human Factor VIII-Related Antigen Established by Hybridization. Proc. Natl. Acad. Sci. 80 (12), 3734–3737. 10.1073/pnas.80.12.3734 6407019 PMC394125

[B11] Gondo Y. Satsu H. Ishimoto Y. Iwamoto T. Shimizu M. (2012). Effect of Taurine on mRNA Expression of Thioredoxin Interacting Protein in Caco-2 Cells. Biochem. Biophysical Res. Commun. 426 (3), 433–437. 10.1016/j.bbrc.2012.08.116 22960072

[B12] Grellier M. Granja P. L. Fricain J.-C. Bidarra S. J. Renard M. Bareille R. (2009). The Effect of the Co-immobilization of Human Osteoprogenitors and Endothelial Cells within Alginate Microspheres on Mineralization in a Bone Defect. Biomaterials 30 (19), 3271–3278. 10.1016/j.biomaterials.2009.02.033 19299013

[B13] Guo Y.-X. Zhang D.-J. Wang H. Xiu Z.-L. Wang L.-X. Xiao H.-B. (2007). Hydrolytic Kinetics of Lithospermic Acid B Extracted from Roots of Salvia Miltiorrhiza. J. Pharm. Biomed. Anal. 43 (2), 435–439. 10.1016/j.jpba.2006.07.046 16950588

[B14] Hui Yu. Xian-Xian Zhao. Xing-Hua Shan. Pan Li. Tao Chen. (2015). Effect of Thioredoxin-Interacting Protein on Wnt/β-Catenin Signaling Pathway and Diabetic Myocardial Infarction. Asian Pac. J. Trop. Med. 11 (8), 95–100.(v. 10.1016/j.apjtm.2015.10.010 26615000

[B15] Jiang Y. Liu C. Chen W. Wang H. Wang C. Na L. (2015). Tetramethylpyrazine Enhances Vascularization and Prevents Osteonecrosis in Steroid-Treated Rats. J. Biomed. Biotechnol. 2015, 315850. 10.1155/2015/315850 PMC4339822 25759816

[B16] Khodneva Y. Shalev A. Frank S. J. Carson A. P. Safford M. M. (2016). Calcium Channel Blocker Use Is Associated with Lower Fasting Serum Glucose Among Adults with Diabetes from the REGARDS Study. Diabetes Res. Clin. Pract. 115, 115–121. 10.1016/j.diabres.2016.01.021 26818894 PMC4887408

[B17] Korn C. Scholz B. Hu J. Srivastava K. Wojtarowicz J. Arnsperger T. (2014). Endothelial Cell-Derived Non-canonical Wnt Ligands Control Vascular Pruning in Angiogenesis. Development 141 (8), 1757–1766. 10.1242/dev.104422 24715464

[B18] Kusumbe A. P. Ramasamy S. K. Adams R. H. (2014). Coupling of Angiogenesis and Osteogenesis by a Specific Vessel Subtype in Bone. Nature 507 (7492), 323–328. 10.1038/nature13145 24646994 PMC4943525

[B19] Lekva T. Ueland T. Bøyum H. Evang J. A. Godang K. Bollerslev J. (2012). TXNIP Is Highly Regulated in Bone Biopsies from Patients with Endogenous Cushing's Syndrome and Related to Bone Turnover. Eur. J. Endocrinol. 166 (6), 1039–1048. 10.1530/eje-11-1082 22450549

[B20] Liao F. (2000). Herbs of Activating Blood Circulation to Remove Blood Stasis. Clin. Hemorheol. Microcirc. 23 (2, 3, 4), 127–131. 11321431

[B21] Luo S. Yang Y. Chen J. Zhong Z. Huang H. Zhang J. (2016). Tanshinol Stimulates Bone Formation and Attenuates Dexamethasone-Induced Inhibition of Osteogenesis in Larval Zebrafish. J. orthopaedic translation 4, 35–45. 10.1016/j.jot.2015.07.002 PMC5986998 30035064

[B22] Maes C. Goossens S. Bartunkova S. Drogat B. Coenegrachts L. Stockmans I. (2010). Increased Skeletal VEGF Enhances β-catenin Activity and Results in Excessively Ossified Bones. EMBO J. 29 (2), 424–441. 10.1038/emboj.2009.361 20010698 PMC2824461

[B23] Mo Y. Lai W. Zhong Y. Hu Z. You M. Du M. (2021). TXNIP Contributes to Bone Loss via Promoting the Mitochondrial Oxidative Phosphorylation during Glucocorticoid-Induced Osteoporosis. Life Sci. 266, 118938. 10.1016/j.lfs.2020.118938 33347878

[B24] Peng Y. Wu S. Li Y. Crane J. L. (2020). Type H Blood Vessels in Bone Modeling and Remodeling. Theranostics 10 (1), 426–436. 10.7150/thno.34126 31903130 PMC6929606

[B25] Phng L.-K. Potente M. Leslie J. D. Babbage J. Nyqvist D. Lobov I. (2009). Nrarp Coordinates Endothelial Notch and Wnt Signaling to Control Vessel Density in Angiogenesis. Dev. Cel. 16 (1), 70–82. 10.1016/j.devcel.2008.12.009 PMC8114544 19154719

[B26] Pignolo R. J. Samsonraj R. M. Law S. F. Wang H. Chandra A. (2019). Targeting Cell Senescence for the Treatment of Age-Related Bone Loss. Curr. Osteoporos. Rep. 17 (2), 70–85. 10.1007/s11914-019-00504-2 30806947

[B27] Prisby R. D. (2017). Mechanical, Hormonal and Metabolic Influences on Blood Vessels, Blood Flow and Bone. J. Endocrinol. 235 (3), R77–R100. 10.1530/JOE-16-0666 28814440 PMC5611884

[B28] Pufe T. Scholz-Ahrens K. E. Franke A. Petersen W. Mentlein R. Varoga D. (2003). The Role of Vascular Endothelial Growth Factor in Glucocorticoid-Induced Bone Loss: Evaluation in a Minipig Model⋆. Bone 33 (6), 869–876. 10.1016/j.bone.2003.08.002 14678846

[B29] Qayyum N. Haseeb M. Kim M. S. Choi S. (2021). Role of Thioredoxin-Interacting Protein in Diseases and its Therapeutic Outlook. Ijms 22 (5), 2754. 10.3390/ijms22052754 33803178 PMC7963165

[B30] Qiu H. Liu W. Lan T. Pan W. Chen X. Wu H. (2018). Salvianolate Reduces Atrial Fibrillation through Suppressing Atrial Interstitial Fibrosis by Inhibiting TGF-β1/Smad2/3 and TXNIP/NLRP3 Inflammasome Signaling Pathways in post-MI Rats. Phytomedicine 51, 255–265. 10.1016/j.phymed.2018.09.238 30466624

[B31] Ramasamy S. K. Kusumbe A. P. Wang L. Adams R. H. (2014). Endothelial Notch Activity Promotes Angiogenesis and Osteogenesis in Bone. Nature 507 (7492), 376–380. 10.1038/nature13146 24647000 PMC4943529

[B32] Reich E. Tamary A. Sionov R. V. Melloul D. (2012). Involvement of Thioredoxin-Interacting Protein (TXNIP) in Glucocorticoid-Mediated Beta Cell Death. Diabetologia 55 (4), 1048–1057. 10.1007/s00125-011-2422-z 22246375

[B33] Sivan U. De Angelis J. Kusumbe A. P. (2019). Role of Angiocrine Signals in Bone Development, Homeostasis and Disease. Open Biol. 9 (10), 190144. 10.1098/rsob.190144 31575330 PMC6833221

[B34] Sivaraj K. K. Adams R. H. (2016). Blood Vessel Formation and Function in Bone. Development 143 (15), 2706–2715. 10.1242/dev.136861 27486231

[B35] Taylor A. D. Saag K. G. (2019). Anabolics in the Management of Glucocorticoid-Induced Osteoporosis: an Evidence-Based Review of Long-Term Safety, Efficacy and Place in Therapy. Ce 14, 41–50. 10.2147/CE.S172820 PMC6711555 31692480

[B36] Thielen L. A. Chen J. Jing G. Moukha-Chafiq O. Xu G. Jo S. (2020). Identification of an Anti-diabetic, Orally Available Small Molecule that Regulates TXNIP Expression and Glucagon Action. Cel Metab. 32 (3), 353–365. 10.1016/j.cmet.2020.07.002 PMC7501995 32726606

[B37] Veeriah V. Zanniti A. Paone R. Chatterjee S. Rucci N. Teti A. (2016). Interleukin-1β, Lipocalin 2 and Nitric Oxide Synthase 2 Are Mechano-Responsive Mediators of Mouse and Human Endothelial Cell-Osteoblast Crosstalk. Sci. Rep. 6, 29880. 10.1038/srep29880 27430980 PMC4949438

[B38] Wang Y. Sang A. Zhu M. Zhang G. Guan H. Ji M. (2016). Tissue Factor Induces VEGF Expression via Activation of the Wnt/β-Catenin Signaling Pathway in ARPE-19 Cells. Mol. Vis. 22, 886–897. 27499609 PMC4961466

[B39] Wang Y. Wan C. Deng L. Liu X. Cao X. Gilbert S. R. (2007). The Hypoxia-Inducible Factor α Pathway Couples Angiogenesis to Osteogenesis during Skeletal Development. J. Clin. Invest. 117 (6), 1616–1626. 10.1172/JCI31581 17549257 PMC1878533

[B40] Wang Z. Rong Y. P. Malone M. H. Davis M. C. Zhong F. Distelhorst C. W. (2006). Thioredoxin-interacting Protein (Txnip) Is a Glucocorticoid-Regulated Primary Response Gene Involved in Mediating Glucocorticoid-Induced Apoptosis. Oncogene 25 (13), 1903–1913. 10.1038/sj.onc.1209218 16301999

[B41] Weinstein R. S. (2010). Glucocorticoids, Osteocytes, and Skeletal Fragility: The Role of Bone Vascularity. Bone 46 (3), 564–570. 10.1016/j.bone.2009.06.030 19591965 PMC2823999

[B42] Wu P. Du G. H. (2015). [Thioredoxin-interacting Protein: a New Potential Target for Diabetes and Related Vascular Complications Therapy]. Yao Xue Xue Bao 50 (12), 1559–1564. 27169277

[B43] Xu R. Yallowitz A. Qin A. Wu Z. Shin D. Y. Kim J.-M. (2018). Targeting Skeletal Endothelium to Ameliorate Bone Loss. Nat. Med. 24 (6), 823–833. 10.1038/s41591-018-0020-z 29785024 PMC5992080

[B44] Yang C. Gu Z. Ding R. Huang C. Li Q. Xie D. (2021). Long Non‐coding RNA MEG3 Silencing and microRNA‐214 Restoration Elevate Osteoprotegerin Expression to Ameliorate Osteoporosis by Limiting TXNIP. J. Cel Mol Med 25 (4), 2025–2039. 10.1111/jcmm.16096 PMC7882928 33393160

[B45] Yang Y.-j. Zhu Z. Wang D.-t. Zhang X.-l. Liu Y.-y. Lai W.-x. (2018). Tanshinol Alleviates Impaired Bone Formation by Inhibiting Adipogenesis via KLF15/PPARγ2 Signaling in GIO Rats. Acta Pharmacol. Sin 39 (4), 633–641. 10.1038/aps.2017.134 29323335 PMC5888681

[B46] Yang Y. Su Y. Wang D. Chen Y. Liu Y. Luo S. (2016). Tanshinol Rescues the Impaired Bone Formation Elicited by Glucocorticoid Involved in KLF15 Pathway. Oxidative Med. Cell Longevity 2016, 1–17. 10.1155/2016/1092746 PMC4808655 27051474

[B47] Yang Y. Su Y. Wang D. Chen Y. Wu T. Li G. (2013). Tanshinol Attenuates the Deleterious Effects of Oxidative Stress on Osteoblastic Differentiation via Wnt/FoxO3a Signaling. Oxidative Med. Cell Longevity 2013, 1–18. 10.1155/2013/351895 PMC3893867 24489983

[B48] Yu H. Zhao X.-X. Shan X.-H. Li P. Chen T. (2015). RETRACTED: Effect of Thioredoxin-Interacting Protein on Wnt/β-Catenin Signaling Pathway and Diabetic Myocardial Infarction. Asian Pac. J. Trop. Med. 8 (11), 976–982. 10.1016/j.apjtm.2015.10.010 26615000

[B49] Zhang H. Shi X. Wang L. Li X. Zheng C. Gao B. (2018). Intramembranous Ossification and Endochondral Ossification Are Impaired Differently between Glucocorticoid-Induced Osteoporosis and Estrogen Deficiency-Induced Osteoporosis. Sci. Rep. 8 (1), 3867. 10.1038/s41598-018-22095-1 29497100 PMC5832871

