# Supplementary material for: Tanshinol Alleviates Microcirculation Disturbance and Impaired Bone Formation by Attenuating TXNIP Signaling in GIO Rats
Source: Front Pharmacol. 2021 Jul 14;12:722175. doi: 10.3389/fphar.2021.722175 (PMC8316650; doi:10.3389/fphar.2021.722175)
Supplement: Supplementary file 1 [file Table1.DOCX]

**Complementary table 1.**

**Table 1. Primers used for quantitative analyses of gene expression**

| Gene |  | Primer sequence |
| --- | --- | --- |
| TXNIP | Sense  antisense | 5’-GGAGGTGTGTGAAGTTACTCGTG-3’  5’-CGTGTCTTCATAGCGCAGGT-3’ |
| VEGF | Sense  antisense | 5’-GCTGACGGACAGACAGACAGA-3’  5’-TCCTCCGAAGCGAGAACAG-3’ |
| VEGFR2 | Sense  antisense | 5’-ACTGACTTGGCCTCGGTCAT-3’  5’-CCGAGACATGGAATCACCAC-3’ |
| CD31 | Sense  antisense | 5’-ACGATGTGGCTTGGAGTCCT-3’  5’-AGTCCGGCAGGCTCTTCAT-3’ |
| HIF-α | Sense  antisense | GCCTCTGTGATGAGGCTTACC  TCACCATCATCTGTGAGAACCA |
| Trx | Sense  antisense | GCAGCCAAGATGGTGAAGCA  CCACGTGGCTGAGAAGTCAA |
| GAPDH | Sense  antisense | 5’-ATTGTCAGCAATGCATCCTG-3’  5’-ATGGACTGTGGATGAGCC-3’ |
