# Supplementary figures and images for: Tanshinol Alleviates Microcirculation Disturbance and Impaired Bone Formation by Attenuating TXNIP Signaling in GIO Rats
Source: Front Pharmacol. 2021 Jul 14;12:722175. doi: 10.3389/fphar.2021.722175 (PMC8316650; doi:10.3389/fphar.2021.722175)

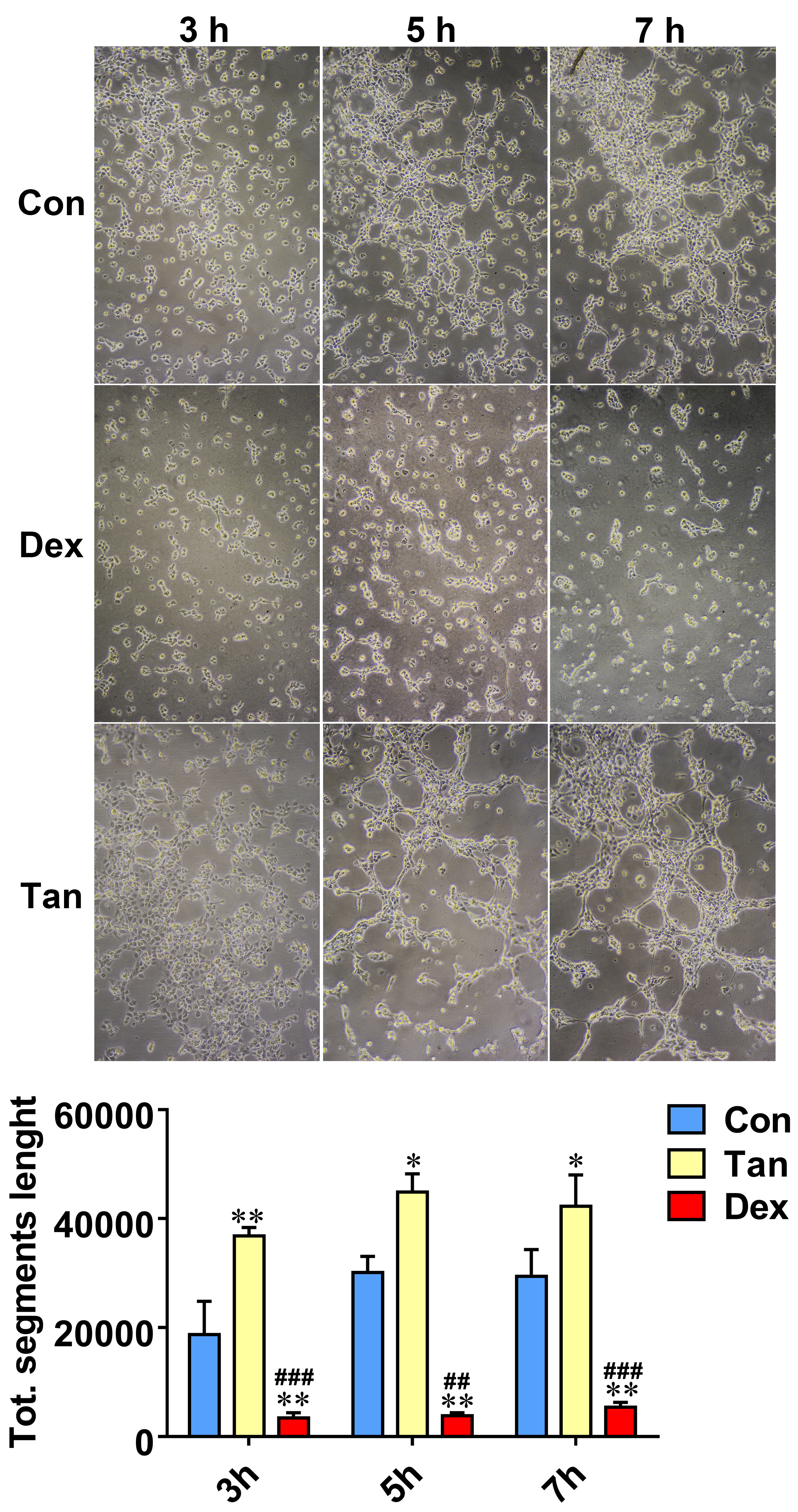

Supplement: Supplementary file 2 [file Image3.TIF]

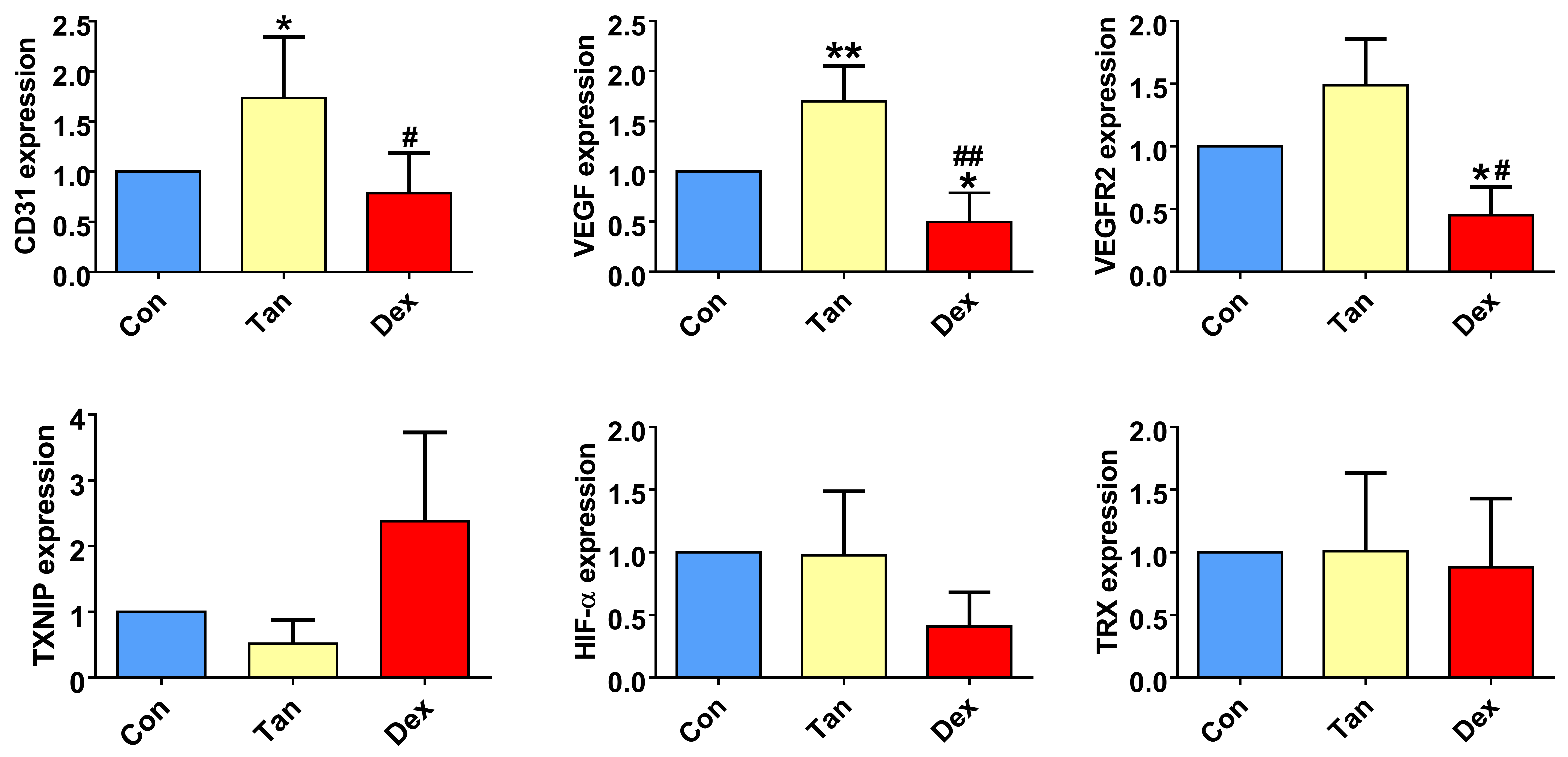

Supplement: Supplementary file 3 [file Image2.TIF]

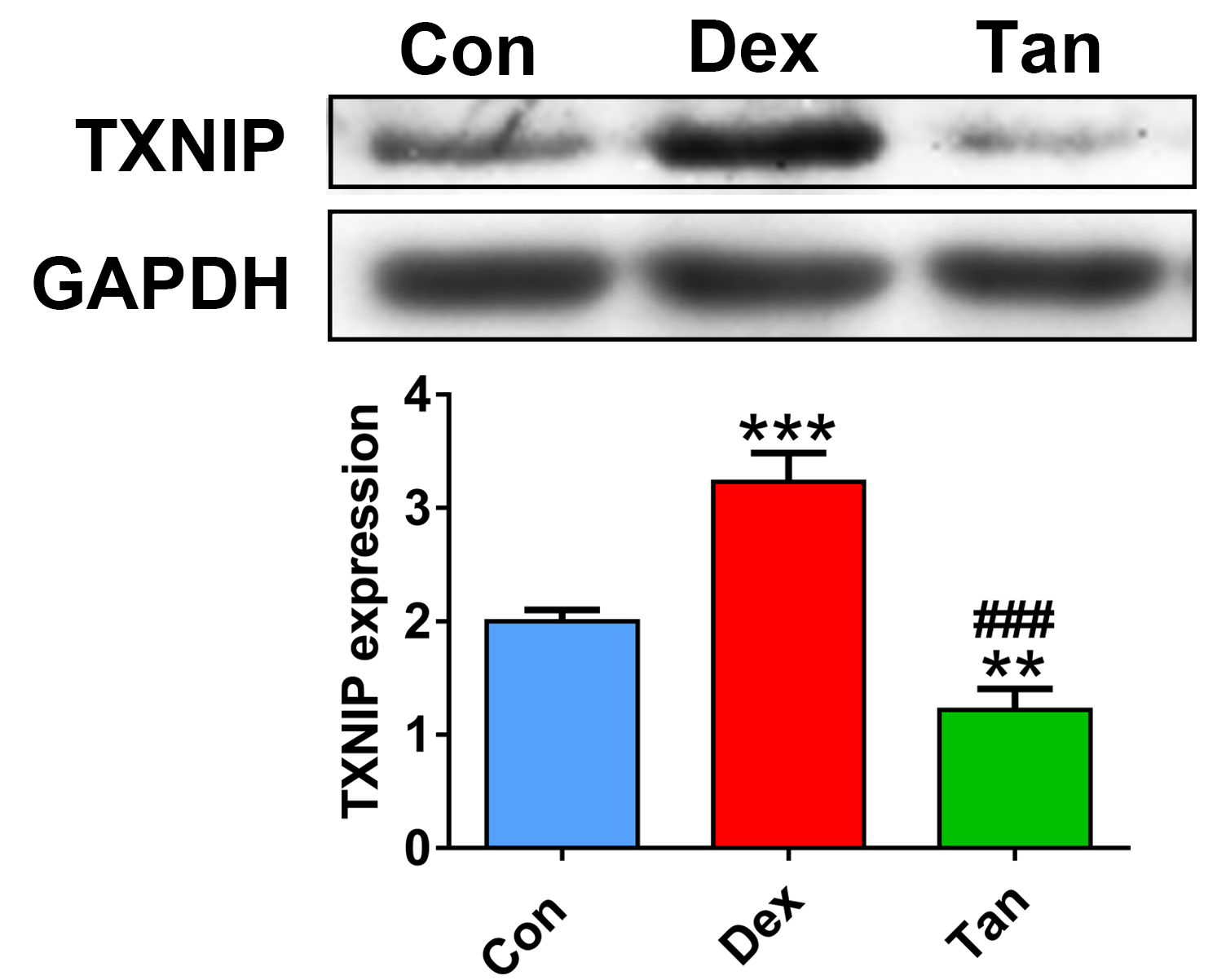

Supplement: Supplementary file 4 [file Image1.TIF]
